# The significance of ethics reflection groups in mental health care: a focus group study among health care professionals

**DOI:** 10.1186/s12910-018-0297-y

**Published:** 2018-06-05

**Authors:** Marit Helene Hem, Bert Molewijk, Elisabeth Gjerberg, Lillian Lillemoen, Reidar Pedersen

**Affiliations:** 1 Centre for Medical Ethics, Institute of Health and Society, Faculty of Medicine, University of Oslo, P.O.Box 1130, Blindern, NO-0318 Oslo, Norway; 2 VID Specialized University, Faculty of Health Studies, Box 184, Vinderen, NO-0319 Oslo, Norway; 3 Department Metamedica, APHVU University medical centre/VUmc), Amsterdam, the Netherlands

**Keywords:** Coercion, Ethics reflection groups, Focus group study, Health care professionals, Mental health care

## Abstract

**Background:**

Professionals within the mental health services face many ethical dilemmas and challenging situations regarding the use of coercion. The purpose of this study was to evaluate the significance of participating in systematic ethics reflection groups focusing on ethical challenges related to coercion.

**Methods:**

In 2013 and 2014, 20 focus group interviews with 127 participants were conducted. The interviews were tape recorded and transcribed verbatim. The analysis is inspired by the concept of ‘bricolage’ which means our approach was inductive.

**Results:**

Most participants report positive experiences with participating in ethics reflection groups: A systematic and well-structured approach to discuss ethical challenges, increased consciousness of formal and informal coercion, a possibility to challenge problematic concepts, attitudes and practices, improved professional competence and confidence, greater trust within the team, more constructive disagreement and room for internal critique, less judgmental reactions and more reasoned approaches, and identification of potential for improvement and alternative courses of action. On several wards, the participation of psychiatrists and psychologists in the reflection groups was missing. The impact of the perceived lack of safety in reflection groups should not be underestimated. Sometimes the method for ethics reflection was utilised in a rigid way. Direct involvement of patients and family was missing.

**Conclusion:**

This focus group study indicates the potential of ethics reflection groups to create a moral space in the workplace that promotes critical, reflective and collaborative moral deliberations. Future research, with other designs and methodologies, is needed to further investigate the impact of ethics reflection groups on improving health care practices.

## Background

### Ethical challenges in mental health care

Professionals within the mental health services face many ethical dilemmas and challenging situations. Prioritising between patients, cooperation between patients and family as well as the use of coercion are important examples. An ethical challenge occurs where there is doubt, uncertainty or disagreement about what is morally good or right [1–3].

Some studies refer to ‘large-scale’ and ‘small-scale’ ethical challenges [4]. A large-scale ethical challenge may be a question concerning whether to put a patient in belts (‘coercion’) [5] while a small-scale ethical challenge may be about whether to reject the patient’s questions about being allowed to call their parents or not (‘persuasion’ or ‘leverage’) [ibid.]. Some distinguish between the terms ‘challenge’ and ‘dilemma’ and by that indicate that a dilemma involves facing a situation where there is no good solution, but where you have to make a choice, typically between two alternatives. We prefer to use these terms interchangeably since an ethical challenge could mean that all solutions have serious downsides.

### The use of coercion in mental health care

Over the last years, the use of coercion in mental health services has received increasing attention. Coercion raises some of the most difficult ethical issues [6]. By coercion we refer to both formal, informal [7] and perceived coercion [8, 9], and between there are many grey areas. ‘Formal coercion’ is formally regulated, decided and documented, while ‘informal coercion’ includes all forms of coercion and use of power, control or manipulation without any formal decision or documentation. ‘Perceived coercion’ may overlap with both formal and informal coercion, and is defined by the individual’s subjective experience of being forced or not.

The use of coercion threatens patients’ autonomy. Coercion can cause psychological and physical harm, and it also threatens health professionals’ perception of what good care and treatment is. Most often, it is used to help the patient. However, it may also be used to protect others or even be misused by professionals. Therefore, the use of coercion, is a complicated moral enterprise.

Szmukler and Appelbaum [5] have developed a hierarchy of pressure that is common in clinical practice, where the lowest level is persuasion (‘persuasion’). The next steps are influence (‘leverage’), request (‘inducements’), and finally threats (‘threats’) that end with the use of coercion, including physical force [ibid.]. The terms ‘coercion context’ [10] and ‘coercive shadow’ [11] show how the coercive dynamics are expressed in different ways in mental health care.

Norvoll and Pedersen [12, 13] show how the ‘coercive shaping’ of mental health care is expressed in hierarchies and communication patterns, the use of house rules, and a paternalistic culture where patients feel the lack of freedom and powerlessness. The experience of losing one’s freedom is a core element of coercion. Patients may feel small, exposed and vulnerable, which may cause them to have difficulty communicating their own needs and desires to staff. The patients’ counter-power strategies can be passive in terms of withdrawal and an attempt to escape or evade contact. Counter-power can also take the form of active resistance, which in turn can cause the patient to be interpreted as ill or lacking insight into his/her own illness. Being coerced may be experienced as an existential event or even dehumanising, since it may influence how the patients perceive themselves and may give rise to a feeling of losing one’s self [ibid.].

### Clinical ethics support related to the use of coercion in mental health care

Over the last decades, health care ethics, laws and policies have given higher priority to patient autonomy, and attempts to reduce coercion and to use coercion in a better way [14]. However, there has been less systematic attention to ethical challenges and how to deal with them - for example how to balance autonomy with beneficence [15] - within the mental health services than in other parts of the health care system [16–18]. It is a paradox since law and practice in mental health services raises many complex ethical challenges (1–6) and probably have the potential to undermine the right to freedom to a greater extent than what is found in any other part of civil society and legislation [19].

In a systematic literature review on the evaluation of clinical ethics support in mental healthcare, the results showed that (a) participants reported that they gained an increased insight into moral issues through systematic reflection; (b) there was improved cooperation among multidisciplinary team members; (c) participants were uncertain whether clinical ethics support led to better patient care; (d) the issue of patient and client participation is complex; and (e) the implementation process is challenging. Clinical ethics support services have mainly been studied through the experiences of the participating facilitators and healthcare professionals. Consequently, there is a lack of knowledge of whether and how various types of clinical ethics support services influence the quality of care and how patients and relatives may evaluate clinical ethics support services. Based on six ‘grey zone articles’, in which there was an implicit focus on ethics reflection, other ways of working with ethical reflection in practice are discussed. Implementing and evaluating clinical ethics support services as approaches to clinical ethics support that are more integrated into the development of good practice was stressed [18].

In a focus group study from municipal health and care services where the aim of the study was to examine what issues the employees discussed in ethics reflection groups (ERG), whether the ethics initiative has had an impact on the quality of the services and work environment, and if so, what kind of impact, and the extent to which it had contributed to increased competence in ethics. Results show that employees of the municipal health and care services experience many complex tasks requiring professional skills, but also situations that require expertise in ethics and health law. Situations involving conflicting value judgments appear to be particularly demanding, and the informants presented situations that challenged them in several ways. The most common topics were the use of coercion, interaction with relatives, and decisions about treatment intensity. The study shows that employees in municipal health and care services find that the ethics initiative has been an important contribution to quality and competence, handling ethical challenges in a better way [20].

In two surveys where municipal contact persons for the Norwegian ethics project and ethics facilitators participated, around half of the respondents found the ethics project to have been highly significant for daily professional practice. Outcomes include better handling of ethical challenges, better employee cooperation, better service quality, and better relations to patients and next of kin. Factors associated with significance of the activities were sufficient support from stakeholders, sufficient available time, and ethics facilitators having sufficient knowledge and skills in ethics and access to supervision. The authors conclude that there is a need to create regional or national structures for follow-up and develop more comprehensive ethics training for ethics facilitators [21].

Sometimes it is obvious which actions and measures are necessary in order to deal with ethical challenges in a good way. Other times it is not clear what the best approach or solution is. Situations can be complex and confusing, which may make it difficult to put into words what is at stake [3]. Systematic ethics reflection can help health professionals develop an ethical language covering the challenges they face. Research shows that reflecting on the challenges faced in daily work, both individually and in the team, may help health professionals to become more conscious of their own understanding, their attitudes and actions [1]. Ethics reflection can contribute to learning, which means finding new solutions, developing a better practice and learning how to work better together [14]. Reflection is arguably an essential feature of all professional development and professional competence [22–24]. Research suggests that ethical reflection groups can help raise awareness about proper use of coercion, alternatives to coercion and better handling of ethical challenges [25–28].

### Presentation of the PET-project

One important exception regarding commitment to systematic ethics work in order to better deal with the ethical challenges related to coercion within mental health care is the Norwegian research- and development project called PET (the English name for ‘PET’ is ‘Mental health care, ethics and coercion’) – running from 2011 to 2016 - which was inspired by Dutch initiatives [1, 25–28] and similar initiatives in other parts of Norwegian health care. The study included four sub studies: a) systematic literature reviews on evaluation of ethics support in mental health care [18] and ethical challenges related to coercion [29], b) interviewing patients, children and next of kin of patients about coercion and involvement [12–14, 30], c) the implementation and evaluation of ethics reflection groups [18] and d) a national survey among mental health care staff and patients on coercion [31]. This article presents evaluation findings from part c.

The PET-project is part of the National Strategy for Increased Voluntariness in Mental Health Care (2012–2015). Mental health care in Norway is publicly funded and organised as ‘specialised health services’ – that is, hospital trusts (hospitals and outpatient clinics) – and as ‘community health services’ (general practitioners, emergency rooms and homecare). Formal coercion mainly takes place within specialised health services, though community health services may request involuntary hospitalisation. The quality of the public health services in Norway is generally high and used by all social classes. Private for-profit mental health services are relatively limited. The PET-project is inspired by discourse/dialogical ethics [32] and addresses the ethical challenges related to coercion and involvement from all stakeholders’ perspectives. However, the project is not about ethical analysis of coercion as such. Ethics reflection groups were offered to employees in order to share their experiences related to the use of coercion and to better deal with ethical challenges related to coercion [2, 3, 16, 25, 26].

This article presents how the participants in the ethics reflection groups evaluate – for good and bad – the significance of these ethics reflection groups.

## Methods

Our main research question was:

- What kind of significance did participating in systematic ethics reflection groups – focusing on ethical challenging coercion and involvement of patient and family - have to mental health care professionals?

In addition, we had questions concerning the implementation and organisation of the ethics reflection groups as well as the training of the facilitators. We will publish a separate article based on the findings from this part of the study.

One of several aims of the sub-project was to establish ethics reflection groups in mental health care as well as evaluating the process. Therefore, regularly occurring ethics reflection groups at seven departments within three hospital trusts in the southeastern part of Norway were established. The participating departments reflect the variety of mental health services: acute, rehabilitation, forensic, adolescent, geriatric and outpatient services. The wards established and conducted ethics reflection groups for two years (2012–2014). All ethics reflection groups were held within the same wards, and within those wards, all (except in one unit) ethics reflection groups were held across units. The Centre for Medical Ethics (CME) supported this initiative by training 21 ethics facilitators who were to lead the groups. Within each ward, one person coordinated the organisation of the ethics reflection groups. Each ethics reflection group was held once or twice a month. A multidisciplinary group of health care professionals (i.e nurses, socio-therapists, psychologists, psychiatrists, doctors, quality management staff, team leaders, managers) participated voluntarily in the groups. Participants were encouraged to bring up their own ethical challenges in connection with the use of coercion. Based on information from 186 facilitator reports, the ethics reflection groups lasted between 50 and 90 min, and the mean and the median numbers of people who participated in the reflection groups was respectively 9,39 and 9,0. The groups were usually facilitated by two facilitators. A stepwise ethics reflection model - the CME-model - was utilised in the deliberations: 1. What is the ethical question? 2. What are the facts? 3. Who are the stakeholders and what are their views? 4. Which values are at stake? 5. Which principles/guidelines/laws are at stake? 6. Which alternatives for action exist? 7. Conclusion. Furthermore, implementation and follow-up was secured from CME (teaching, supervision, network meetings), as well as research on several areas (ethical challenges, team work, coercion, inclusion of patients and network, ethics reflection groups) utilising a broad range of research methods (questionnaires, focus group interviews, facilitator forms). More details about the organisation, implementation and functioning of the ethics reflection groups, as well as the training of the facilitators, will be presented in another paper [33].

### Focus group interviews

The rationale for conducting focus group interviews was that we wanted to talk to a large multidisciplinary group of health care professionals in order to cover as wide a range of experiences with ethics reflection groups as possible. In addition, our aim was to learn about the interpersonal dynamics and culture while health care professionals talked about doing systematic ethics reflection [34–40]. Consequently, in 2013 (after one year of running ethics reflection groups), we performed 13 focus group interviews; seven focus groups with 53 clinicians and six focus groups with 32 members of the management. In 2014 (after two year of running ethics reflection groups), we conducted seven focus group interviews with a total of 42 participants (combining clinicians and management). In total, we conducted 20 focus group interviews with 127 participants (some participants took part both years). The reason for the high numbers was that we wanted to have a good view of the experiences with the ethics reflection groups for each ward. In addition, the focus group interviews functioned as a way of staying in touch with the seven wards and facilitating their implementation process (i.e. the focus group interviews did not only function as data-collection). Furthermore, the planned focus group interviews created an opportunity for the participants to reflect together on the implementation of the ethics reflection groups (which most teams otherwise would not have planned by themselves). The focus group participants were not the same as the ethics reflection group participants. We asked the local coordinators of the ethics reflection groups to take responsibility for organising the focus group interviews. They put together participants for the focus group interviews based on what was practically possible to accomplish (due to people being busy on the ward, off duty, on sick leave or on holidays).

Focus group interviews are usually conducted by a moderator who will safeguard that all voices are heard, that the dialogue is based on the subject that is in focus, and that the group’s experiences are expressed through the conversation [34–40]. We chose to have two moderators (first and second author), and we supplemented each other with questions. Furthermore, since moderators must also be attentive to the group dynamics [35, 40], we were conscious about creating an accepting atmosphere so that the participants would feel free to talk [35]. We followed up with questions for elaboration.

We explicitly invited more and less experienced ethics reflection group participants. We also invited participants who were positive as well as critical towards ethics reflection groups. In the beginning of the focus group interviews, we explicitly stated that we would like them to share both positive and critical experiences with the ethics reflection groups. In addition, we explicitly instructed the participants not to focus on consensus but learn from different perspectives on the subject (as usually happens within ethics reflection groups). Furthermore, we wanted to include people from the management in the focus groups, since we assumed that they would offer nuanced and more distanced views on positive as well as negative experiences with the ethics reflection groups.

The interviews were tape recorded and transcribed verbatim, and consist of 200 pages (2013) + 195 pages (2014), in total 395 pages.

### Data analysis

The analysis is inspired by the concept of ‘bricolage’ [41, 42], which means we have moved freely back and forth in the data material. Our approach was inductive [42, 43]. First, all five authors did a naïve reading of all the transcripts in order to obtain a first and overall impression of the data material. Each one of us, independently, made a rough outline of what we found interesting and important. This formed the basis of our first discussions. We proceeded by starting to make categories based on our impression of the material and agreeing on the main findings. This led to the initial structuring of the material by themes. We made an overview of descriptions involving experiences with ethics reflection groups, which formed the central meaning units. The empirical material was reread several times in order to validate the main categories which are described in the results section of this paper: their experiences with systematic reflection on ethical challenges, increased awareness of the use of coercion, improved interdisciplinary cooperation, and lack of involvement of patient and family.

We have discussed with each other by sharing our thoughts and impressions, and we have basically agreed on the interpretation of the findings. This means that we have exploited the fact that we are five researchers with both similar and dissimilar theoretical and empirical interests; as a whole we possess comprehensive knowledge of doing systematic ethics reflection in health care, as well as extensive experience utilising qualitative research methods. Likewise, we have presented preliminary findings to health care professionals and researchers, which contributed elaborations, amplifications and clarifications of the results. Through this work we have aimed at meeting both primary (credibility, authenticity, criticality, and integrity) and secondary (explicitness, vividness, creativity, thoroughness, congruence, and sensitivity) criteria of validity [44].

### Ethical considerations

The work was undertaken conforming to the provisions of the Declaration of Helsinki [45], which means that basic ethical principles for research ethics such as informed consent, the right to privacy, respect for personal integrity and dignity [41, 45] were followed. To protect the patients’ privacy, we asked participants in advance to mask characteristics that may contribute to recognition. All participants gave informed consent after having received written and oral information about the project. The information contained, among other things: the aims and topics of the focus group interviews, the confidentiality of the data, the way we will store the data and use the analysis for scientific papers and presentations, and the possibility to withdraw from the focus group study at any moment without giving any reason. During the transcription process, we have also been mindful to change the names of people, institutions, and places, as well as considering all information with regard to the risk of identification of individuals.

## Results

In the PET-project, we have studied what kind of ethical challenges the professionals report that they struggle with in their daily practice. They experience ethical challenges in areas like the use of formal as well as informal coercion, that they feel insecure about the legal framework for coercion, their identity and professional role, that they experience organisational difficulties and lack of resources, and the abuse of power and lack of professional competence among some colleagues [46, 47]. The professionals experience challenges, which seem to occur across various wards and services, as well as challenges that are context specific [29]. The present study shows that systematic ethics reflection makes a difference for the professionals’ perception of and dealing with ethical challenges related to coercion in several ways.

### Experiences from systematic reflection on ethical challenges

#### The structured model for reflection is helpful

Most of the participants value the systematic way of dealing with ethical challenges (the CME-model for ethics reflection), and appreciated the CME-model which was used in the reflection groups. In some places, the systematic approach to ethical challenges has become part of the ‘culture’, meaning that they try to or prefer to approach various (ethical) challenges in a more systematic way. This means that they recognise the ethical challenges and speak about them in a structured way, which they did not do before they implemented ethics reflection groups. The CME-model is fixed, and serves as a guide for how to move ahead with the reflection. It safeguards a systematic and well-structured approach to talk about ethical challenges, which again allows space for everybody participating in the group. The dialogue between the participants allows for multiple perspectives on the ethical challenge.

Furthermore, the attention to values and norms in the CME-model, and the invitation to the participants to present their viewpoints, contributes to the understanding of those who think differently, for instance regarding the use of coercion. Also, the focus on various alternatives for dealing with the ethical challenges has been evaluated positively by most of the participants. However, the model in itself does not guarantee a successful reflection group. It is important to use the model in the right way, and people may think differently about what the right use of the model is. For example, it seems to be important that the use of the model does not become too rigid. On one of the participating wards, they found that an inflexible use of the model hampered the natural flow of the reflection process: participants sometimes could not contribute to the reflection because the facilitator said they had not yet arrived at this point in the reflection model. Hence, they found that the facilitator managed the model in a linear way. Another participant mentioned that the facilitator had too much focus on where to put the points for reflection in the model, leading to more attention on how to operate the model than on the content of the case.

Most participants describe that they have moved from having ‘opinions’ or emotional reactions concerning the situation at hand, towards being able to provide reasons and work analytically with the ethical challenges they face. This ‘move’ increases their consciousness and it safeguards a more structured approach to morally demanding situations. One said:

I find that our way of talking with each other is different from when we have meetings where we discuss patient treatment. People are calmer … people are not so emotional. I think we share difficult issues in a different way in those meetings.

The step in the model dealing with ‘alternative course of action’ is helpful since it illuminates that there are several options or ways to proceed in a certain situation. Several participants said that using the model can be helpful in discussions about subjects other than coercion as well, and that they had used the model in other meetings discussing patient treatment. One said it this way: *I find that I am aware of the different stages of the model, I am conscious of them, and they pop up in other situations, as well*.

#### Professional development and better quality of treatment

Regarding ethics reflection, some say they think the most important thing is to be ‘in process’, which means the adoption of an attitude where sharing different opinions and viewpoints among colleagues is a value in itself. They acknowledge that they are now able to reflect more than before; some said they used to act in a more reactive manner before they started with the ethics reflection groups. Their enhanced ability to reflect makes them feel more secure as professionals. One said:

The first step towards change is through acknowledging the use of coercion as an integrated part of a culture which is accustomed to setting limits for others. Through ethics reflection groups, our language is challenged concerning what we are actually doing. Our concepts and the way we use them are being questioned.

Many participants report that the deliberations in the reflection groups have helped them to develop a language describing the ethical challenges they face. Participating in such deliberations makes health professionals more experienced in identifying ethical challenges. One participant used the word *character formation*, feeling that the deliberations they have in the ethics reflection groups create professional development among colleagues over time. This participant indicates that some think their attitudes are changing from being rigid and inflexible to becoming more open and letting themselves to a lesser extent be governed by formal rules or old habits. The participants say that they have developed an increased awareness about the fact that there is not always one solution or one concrete answer in a situation. They report that they have developed a space for reflection where it is possible to ask questions like: *What could I have done differently*? Consequently, they are – more than before - able to see alternatives. Furthermore, several of them say *It is important to reflect on how exercising coercion is affecting me*. They find they have developed a stronger competency to make more correct decisions, which again is increasing their professional competence and confidence and the quality of treatment. *Keeping up reflecting creates better treatment*, one participant said.

Moreover, reflecting on ethical challenges together, they report, has led them to greater tolerance for different opinions than before, which again creates a stronger ability to be self-critical. They have gained a broader perspective, they think their attitudes have changed and they see the value in illuminating different opinions, or utilising each other’s perspectives and competence.

#### Dealing with emotions in ethics reflection

Health care professionals are often an affected party in clinical situations, and there is room for sharing this (referring to ‘affected parties’ in the reflection model). They tell us that they receive help in sorting through difficult feelings, and they acknowledge that everybody has a lot to learn. They say that the CME-model is a support when it comes to adopting a critical/analytical stance towards their emotions. They think that staying focused on the solutions in a situation (“How are we going to deal with this dilemma?”) helps them to not be overwhelmed by their feelings.

They feel that connecting emotions to the values at stake is a different way of framing and containing emotions, which they appreciated.

People can express their emotions, but it is different, the dialogue is framed in a way so that they do not accuse their colleagues or get overwhelmed by their feelings. We can talk about emotions, reflect, and connect them to values.

Furthermore, some said that reflections focusing only on emotions were of lesser quality. Several of the participants on this ward were critical of ethics reflection groups because they felt they needed theoretically informed discussions on ethical challenges, and not reflection on clinical cases and emotions connected to working with them. They thought that as clinicians they would profit from gaining more knowledge about normative theories. In addition, some participants on another ward - after having participated in ethics reflection groups - found that they became anxious about making mistakes in the treatment of the patients. Hence, they could recognise that they had a tendency before to act in situations, instead of sometimes stepping back and reflecting critically on what would be the best way to approach the situation. Acting without reflecting could for instance lead to unethical behaviour, like putting the patient in belts. However, being afraid of behaving in an unethical manner could result in interfering too late, e.g. in one crisis situation, the patient could lose control and act out. After one such incident, they needed to work on how to balance between interfering and not interfering, and search for alternative ways of interfering.

### Increased awareness of the use of coercion

The participants talk about having developed a critical attitude to, and an increased awareness, about their use of coercion. They say they are now reflecting and asking more questions about their own practice. Their thinking is more focused so that the use of coercion does not become a routine. Ethics reflection contributes to the health care professionals’ staying on top of the situations, meaning that they are proactive, which they say is an indicator of good quality. Consequently, they are able to raise new and self-critical questions like *What are we actually doing*?

#### Increased awareness of the use of informal coercion

Increased awareness also resulted in acknowledging that the use of coercion is located on a continuum: on the one side of the continuum there is the use of power and informal coercion (often invisible or subtle, like using manipulation or pressure) and on the other side you find formal coercion (often visible and concrete, like using belts or forced medication). Taking part in ethics reflection groups has resulted in the health care professionals becoming conscious of the power they possess, and their use of informal coercion. They ask themselves questions about “how they coerce” like:

How do we welcome a patient? How do we approach patient and family when they show up at the acute unit in a crisis? How do we care for them? Do we offer something to drink? Are we nice, and do we inform them about the reason why we have to go through the patient’s luggage?

Along these lines, realising there is an effect on the ‘small-scale’ phenomena, they also contend that they have developed increased focus on “more correct” use of coercion and alternatives to coercion: *What kind of limitations should we put on this patient in this situation? For how long should she be ‘shielded’? How long should the patient be allowed to be outside the ward?* They focus critically on their daily routines: *We have discussions about how long people should be allowed to stay in bed; where, when should we interfere, what do we do?*

#### Awareness of the terms ‘us’ and ‘them’

On some wards, there had been a tendency to draw a strict line between “us” and “them” (i.e. us as professionals and them as patients). The participants described an increased awareness concerning the potential negative effects of this kind of categorisation, and about what you are talking about when the patients are present. One example was to share with colleagues what you are going to do the coming weekend or during holidays, something that can be seen as innocent small-talk or as a conscious or subconscious attempt to make the differing situations of patients and professionals utterly visible.

*Even if you do not think about it, there is a tendency in our attitude that “I have and you have not, I can leave at 3 pm., you have to stay. I go to the mountains on Friday, you get pizza or porridge tomorrow*”.

In other words, the critical view in the reflection groups, including the topic of everyday activities, made visible aspects they did not think about because they belonged to the everyday routines of the ward. The more extreme cases - connected to formal use of coercion - are more defined and clear, which the everyday challenges are not, they contend.

#### Cooperation rather than coercion

Participants say they are changing their perspective by starting to ask themselves why coercion is necessary. They tell us that sometimes – after having had ethics reflection - there was no need for coercion any longer. Furthermore, they think that systematic ethics reflection is informative for clinical practice in the way that it fosters cooperation with the patient: they now more systematically initiate dialogues with the patient, for instance about how he or she experienced coercion and informing the patient about their own views and reasons. Sometimes, the results from systematic ethics reflection were written in the patient’s chart, which led to a change in their approach towards the patient, or the coercion that is being used, or the way the coercion is being executed. Participants from one ward told us that one of their male patients was severely and repeatedly degrading female staff members:

The patient, who was very hostile towards the female staff, treated them in a disgusting way. He really crossed the line. The female staff kept him at four arm’s length. Through ethical reflection, however, they managed to change their attitude towards him. They decided to start being nice to him. They said to themselves: “Whatever you feel now, approach him in a friendly way.” The result was that the patient also changed his way of being towards the staff.

When the staff perceived this patient as disrespectful towards them, it is easy to imagine that they would be unfriendly and possibly be more inclined to use informal or formal coercion in their interaction with him. One can assume how such a way of interacting could develop into a ‘coercive relationship’ rather than a cooperative one.

Several participants felt that the ethics reflection groups contributed to a better understanding of the perspectives of the patient and the relatives. The deliberation method used in the ethics reflection groups encourages the professionals to identify all stakeholders and examine their views, i.e. both the professionals, patients and the family. Furthermore, the topic of many dilemmas was how to involve the patient and/or the family and prevent coercion. One person said: *The patients and family members are described in a very respectful way in the reflection groups. I think this leads to better involvement of patient and family in our clinical work*. Some participants were unsure about this possible effect, since they had attempted to involve the patient and family before they started to run ethics reflection groups.

### Improved interdisciplinary cooperation

#### Reflecting together

In general, many participants appreciate the fact that the reflection groups created an explicit and structured area for reflection, both among their colleagues and among various professional disciplines. In addition, some participants state that for them the reflection groups are the only democratic arena at the workplace. Taking part in the ethics reflection groups removes the hierarchies between professionals due to the focus on everybody’s own ethical reflections. More in general, many participants highlighted the values that were practiced within the ethics reflection groups: equality, respect, active listening, taking the perspective of the other, and speaking freely without being personally criticised. They acknowledge that through the ethics reflection groups they realised they were not alone in experiencing an ethical challenge. Creating a culture where difficult dilemmas are deliberated among various disciplines is of great value, leading to a collective basis for decision-making. This often led to the shared understanding that *The conclusion is owned by everybody*.

On one ward, several participants claimed they did not profit from ethics reflection groups since they thought their discussions about ethical challenges already were on a high level. This was in contrast to the level of the deliberations in the ethics reflection groups, as they experienced it. They thought that the exchanges were emotionally laden at the expense of the health care professionals’ cognitive capabilities. Sometimes, the cases presented were on the extreme side and not really relevant, they wanted more focus on everyday problems. On this ward, there were several psychologists being trained as clinical experts, and they felt their needs for supervision and reflection were well taken care of in the programs they were enrolled in.

#### Building a professional culture

By elaborating on dilemmas and by challenging people’s basic assumptions, systematic ethics reflection is a way to build a professional culture. One person was explicit, saying that they wanted to *create a culture where we talk about challenges as a team*. Learning from each other contributes to teambuilding and a broader foundation for cooperation. By creating common perspectives, which is demanding because they came from different backgrounds and different professional cultures, they became closer as colleagues. It was a way of creating trust in the team, and hence they became more effective as a team, enabling them to offer better treatment to the patient.

Having reflection groups across wards, which most of the participating units had, meant they got views from outside, creating new perspectives. Learning from each other *is like seeing the elephant from different angles*, one participant said. By this, they also learn about the different cultures existing on different wards and departments. In addition, they say it is important to arrange group reflection after a difficult incident, also to talk about disagreement that might have occurred. However, showing feelings in reflection groups normally requires that one feels safe with the colleagues, some point out. Not knowing each other and having large groups can compromise feelings of safety, which may have an impact on the quality of the ethics reflection. Some employees at one unit felt criticised and misunderstood by colleagues from another unit, and they ended up defending themselves. They also tell us that they were frustrated because they spent a lot of time explaining challenges in their work, since the other participants did not know the details of their work situation. They felt that defending and explaining took time and energy away from concentrating on deliberating on the ethical challenge they were facing. The employees on that unit needed to find a way of doing ethics reflection that felt constructive and not destructive, which meant that they gave up having ethics reflection together with another unit. They created two subgroups.

On several wards, the participation of psychiatrists and psychologists was missing. Several participants said that psychiatrists and psychologists – because they have the final decision-making responsibility in patient treatment - contribute with a different perspective than the nursing staff, who participate the most in the groups. Many said this is a problem, due to the need to develop a greater understanding of each other’s perspectives. On a couple of wards, they had, for that reason, decided to make it obligatory for the professionals who have formal responsibility for the patient’s treatment or care to take part in the ethics reflection groups. This resulted in greater safety for everybody in establishing a common ground for patient treatment.

Several participants reported on the fact that some of the professionals attending the reflection groups did not actively take part in the discussions. They were perceived as passive and just listening. Therefore, in those refection groups, it was often the same people initiating discussions, i.e. they presented questions and topics and followed up in the deliberations during the group session. Hence, the professionals in such groups were not able to profit from each other’s competence as much as they would like to or could have done. Consequently, according to these participants, the fostering of a professional culture was compromised.

### Lack of direct involvement of patient and family

Even though the health care professionals perceived that the inclusion of the patient and the family’s perspectives was improved in clinical work following the implementation of the ethics reflection groups, the patient and the relatives were not physically present in the deliberations in the groups. Generally, the participants did not talk much about involving the patient and family directly in the ethics reflection groups and no one reported having tried to invite them to the groups.

## Discussion

This focus group study shows how mental health care practitioners describe their experiences from two years of ethics reflection groups. In summary, most of the participants report positive experiences. The positive effects of the groups include: A systematic and well-structured approach to discussing ethical challenges, a space where all professionals can participate, multiple perspectives, improved professional competence and confidence, consciousness of formal and informal coercion, constructive disagreement, truly learning from each other, creating trust in the team, better understanding of those who think differently, a challenge to paternalistic and coercive concepts, attitudes and practices, less judgmental and emotional reactions, acknowledging and dealing more constructively with the stakeholders’ emotions, more analytic and reasoned approaches, room for internal critique and identifying potential for improvement and better alternative course of action. The deliberations and methods used in ethics reflection groups are also reported to influence the clinical work in general, e.g. better interdisciplinary cooperation and inclusion of patients’ and relatives’ perspectives. However, participants also had critical remarks regarding ethics reflection groups, i.e. lack of flexibility in the way the reflection model was used, too much or too little emphasis on emotions, not sufficient focus on normative theories in the deliberations, insufficient interdisciplinary composition of the groups, and lack of direct involvement of patients and family.

In the following discussion, we will first present some strengths and limitations of our study, and then we will discuss some of the main results.

### Strengths and limitations

Those participants in the focus group interviews who had been actively engaged in the discussions in the ethics reflection groups reported that they had positive experiences. However, there were also employees who had not participated in ethics reflection groups or participated only once or a few times. Although these participants lacked extensive experience with the ethics reflection groups and its significance, we think the varied experiences and levels of experience are important when wanting to explore the possible significance of ethics reflection groups.

One strength of this study is that it provides many possible reasons why ethics reflection groups are regarded as positive or not. In this article, we have relied on only one source of data, namely two rounds of focus group interviews. However, we have also evaluated the usefulness and outcomes of ethics reflection groups via various validated and self-developed questionnaires. Preliminary analysis from these other studies, which will be published later [33], corroborates with most of the findings of this study. If we had done extensive observational studies in addition, our data would possibly be even more differentiated for instance regarding the use of informal coercion and the contribution of ethics reflection groups to improved team cooperation. However, we think that the focus group discussions stimulated critical exchanges between the participants. We tried to invite those who were more critical, as well, but they were clearly in the minority in the focus groups. The major strengths of this study is that it includes many participants, different types of wards, two series of interviews after one and two years of the intervention, the use of multiple methods, and that the findings are consistent across the interviews and methods used. This study supports the claim that group reflection can be beneficial through exposing people to different points of view but it also offers more detail about how it does this in the specific context of mental health care and through the specific framing of ethics reflection groups.

Finally, we as interviewers also did the training and represented the group of researchers involved in the intervention study, which may have created bias by influencing the group discussions, and our interpretation of the results (e.g. by being less critical even though we explicitly asked for critical evaluations of the ethics reflection groups as well). On the one hand, we believe that this is part of a complex real-world research environment, but, on the other hand, we might not have sufficiently acknowledged the importance of stepping back from investment in the process as advocates for it. However, implementation research can be considered a “hybrid construction” since it is useful for the construction of knowledge, as well as having a normative agenda: helping wards with implementing ethics reflection groups and improving coercion practices. This requires balancing several competing positions.

### Improved quality of the use of coercion through dealing better with ethical challenges related to the use of coercion

The participants in this study report that the ethics reflection group may have contributed to the reduction of the use of coercive measures on the one hand and an improvement of the use of coercive measures on the other hand. The participants also reported some examples in which the impact had been experienced after the actual deliberation and the concrete dilemmas dealt with in the groups.

In the ethics reflection groups, the professionals reflected upon ethical challenges related to the use of coercion. ‘Coercion’ was broadly defined: we asked them to include formal, informal and perceived coercion. We did this on purpose, since the literature [7–9] and our own research indicate that coercion in mental health care is a complicated ‘moral enterprise’, encompassing both ‘big moral dilemmas’ (e.g. forced medication or physical restraints) and ‘small everyday moral issues’ (e.g. related to asymmetric relationships, pressure, communication and cooperation) [3, 16, 29]. Furthermore, they were asked to use a systematic approach (the CME-model).

The health professionals participating in this study report that they have developed an increased awareness of the way they exercise coercion – not limited to the cases deliberated in the groups. They relate this change broadly to the ‘coercive culture’ - or ‘coercion context’ [10] - within which they operate. The participants also strongly underscored the importance of reflecting on informal coercion. Many talked about how they and their colleagues – during the two-year project period - developed an increased awareness of ethical challenges related to informal use of coercion. They had been able to develop both an awareness regarding recognising ethical challenges, and a moral language with which they could make the previously implicit ethical challenges more explicit. This is promising “since many of the most frequently experienced ethical challenges are not given much notice in traditional medical and health science ethics and are not even regarded as ethics by many” [2, 104]. Ethics reflection groups seem to have the potential a) to analyse and challenge habitual ways of thinking, talking, acting and reacting, b) to identify challenges and potential for improvement, new and better solutions, and c) contributing to changing and improving certain routines and ways of thinking, without causing insurmountable resistance.

Systematic ethics reflection is not a top-down enterprise in which the health professionals are told that they should think and act in a certain way. Rather, it is a bottom-up approach to change through professional growth, internal deliberations, and interprofessional learning which is regarded as a safe, respectful and inspiring start towards improvement [48, 49]. Health care professionals generally bring with them diverse expertise and experiences. This may be a big challenge when interdisciplinary teamwork or cooperation across the wards is required. However, this pluralism in perspectives may also represent a huge potential for mutual learning and quality improvement if disagreement and alternative solutions are identified and dealt with constructively [23, 48, 49].

Another possible contribution to the improvement of the quality of coercion relates to taking into account the perspectives of the patients and next-of-kin when coercion is at stake. Research has shown that patients and next-of-kin regard the use of coercion – in particular the use of informal coercion - as problematic, and they find it important to be involved in the decision-making processes to a greater extent than is the case today [12, 13, 50]. The fact that health professionals - through systematic ethics reflection – are encouraged to identify and describe the view of all stakeholders may represent a small but important step in services where this is often not done. It may also be one important strategy in creating a culture that is less ‘coercive’ and more inclusive. According to Kierkegaard [51], it is easier to help in a good way if health professionals understand the perspective of the person they want to help. Similarly, the Norwegian philosopher Vetlesen [52] contends that moral judgment and helping action is only possible if we understand what is at stake for the other. This may sound like self-evident or superfluous insights. However, interviews with patients and relatives on coercion and involvement indicate that these ‘basics’ are often missing in mental health care, when the patient is severely ill [12, 13, 50].

Even though participants clearly reported that ethics reflection groups contributed to changing their attitudes and ways of thinking about coercion, we should be cautious regarding the causal relationship between reflection groups or moral case deliberation and the improvement of quality of care (e.g. through reduction of the use of coercive measures). Two international studies have reported positive results due to case discussion, clinical case review or facilitated deliberation. Donat [53] found that there was a reduction of use of seclusion and restraint after the use of clinical case reviews and identifying critical cases. Furthermore, Gaskin et al. [54] found that staff integration, treatment plans and treating patients as active participants improved through meetings being conducted with an outside facilitator to analyse the root causes of ward issues and to produce possible solutions. Yet, despite these findings, the lack of causal relationship between (any) interventions and the reduction of coercion, has been stressed recently when looking at the use of appropriate research designs. Van de Sande et al. [55] describes a Cochrane review [56] covering 2155 citations which found no randomised controlled study investigating the effects of interventions aiming at reducing seclusion. Likewise, a more recent review by Stewart et al. [57] could not identify well designed studies in this domain since 2000.

### Critique and safety – A delicate balance

Safety is an important requirement in order to create an atmosphere in which one dares to reflect on challenges regarding coercion. Furthermore, being willing to look at your way of exercising informal coercion – which we have seen has been important for the participants in this study – might be threatening since it may be connected to the way you use your personality as a professional [58]. Reflecting on ethical issues inherently involves asking (self)critical questions. For some, this can be threatening while for others it does not cause such feelings. Generally, participants in an ethics reflection group need to feel safe enough in order to (among other things) reveal that one does not know what the right thing to do is, to share emotions, and to disagree with others. Such a process of moral change through dialogue is described by Landeweer et al. [1].

We found that not knowing each other and/or large ethics reflection groups (e.g. 18 participants) might compromise the feeling of safety, especially where the groups consist of people from different units with the same ward. As we have described, some health care professionals from one ward tended to feel criticised by the questioning of participants from other wards. The reason for some participants feeling criticised and consequently withdrawing from cross-ward groups might be that those participants were feeling especially vulnerable and insecure (maybe regardless of the ethics reflection groups). Another way of understanding this is that the content of the questions, or the way the questions were asked, was too critical and maybe too provocative for these participants. Most likely, it is a combination of the two.

It is important that the group atmosphere is characterised by mutual respect, openness and good will. However, what is enough respect, openness and good will cannot be defined in advance; we probably need a flexible approach responding to what is happening and what people experience. We do not want to say that one should not be critical, but it is important to balance this against the need for safety, so that the participants perceive the ethics reflection groups as something positive, adding value and quality to their way of performing their job. One could, for instance, be open and curious when asking questions rather than being judgmental or confronting. On the other hand, a professional should aim for willingness to be self-critical, receiving challenging questions, and learning from colleagues. Mann et al. [23] suggest that group reflection (“shared reflection”) can be beneficial through exposing people to different points of view. Discussing the question whether reflective practice can be taught and learned, they say: “The factors [that contribute to] … appear to be a facilitating context, a safe atmosphere, mentorship and supervision, peer support and time to reflect” [ibid., 614].

Creating safety and at the same time stimulating critical reflection remains an ongoing tender balance, though, and here is where the facilitator has a vital role in manoeuvring between safety and critical exploration, realising that without feeling sufficiently safe, people may restrict themselves in opening up and in scrutinising how they feel, think and act. In the latter case, facilitators should be aware of this tension between freedom of speech and critical questioning, and at the same time not making the participants feel insecure. This requires not only skills and tact from the facilitator, but in the long run, also from every team member.

How to raise critical questions in a constructive way, without undermining safety and curiosity in the groups, is an important area for further research.

### The role of emotions in ethics reflection groups

The CME-model used in this project does not put special emphasis on emotions. Moral deliberation in many forms – including the CME-model – could be criticised for focusing only on rational arguments and for being too cognitivist. Thus we were surprised that so many of the participants appreciated the way emotions – sometimes strong emotions – were taken care of and framed within the CME-model, despite the model’s rather rational framework. It appears to be common – and regarded as necessary - to share emotions among colleagues when working with people with mental health problems, and many professionals have been trained specifically to handle their own and the patient’s emotions in clinical work. For example, within psychodynamic approaches to clinical work, it is emphasised that professionals’ emotions may carry valuable information concerning what is at stake for the patient and for the professionals (transference and countertransference [59]), and that a general key to high quality treatment (across many different types of therapeutic approaches) is that the professionals are able to develop trusting and safe relationships with the patient and the family. Furthermore, the professionals’ ability to emotionally self-regulate is by many regarded as one of the most important and basic requirements [60].

According to this kind of approach – if emotional reactions are not handled in a competent way in the professional team – there is the danger of displaying negative reactions and distance to the patient. Thus, being reactive - meaning acting on one’s emotions rather than acknowledging emotions and reflecting on their meaning - may have adverse effects in clinical work. Framing one’s emotions within a structured model in systematic ethics reflection – through describing the ‘involved’ or ‘affected’ parties and their views, interests and experiences - appears to be a possible way of dealing more constructively and analytically with emotions. The fact that the participants were so content with this way of working with emotions, might indicate that they realised that their emotions were treated more respectfully when included in a structured and thorough deliberation where many aspects of the ethical problem were included. Furthermore, emotions are important for both detecting ethical challenges and reflecting upon what is at stake in the situation [52]. Hence, emotions are connected to reasoning, and in that way, emotions serve the moral inquiry. They are not taken for granted, neither are they neglected, but are questioned in order to develop a better understanding of the moral issue at stake [61, 62]. To train one’s sensitivity to ethically important moments in clinical work is termed ‘ethical mindfulness’ by Guillemin and Gillam [4, 58].

### Involving patient and family in ethics reflection groups

Some participants seem to think that participating in the ethics reflection groups seem to improve patient and family involvement in clinical work. However, the participants tended to become rather vague when we asked about involvement of patient and family in the ethics reflection groups. Mostly, they were not yet prepared to involve them directly in the ethics reflection groups. However, they said that the fact that the CME-model explicitly asks about ‘involved parties’ was inspiring. Some said it urged them to involve the perspectives of patient and family to a greater extent in their deliberations and in their clinical work. Nevertheless, there may be a potential to make ethics support even more democratic or inclusive, and to learn more through involving the patient and family directly in the actual ethics reflection groups [49, 63, 64].

There are different ways to include the patient and families. The most direct is to include patient and/or a family member in the group deliberations. Another way is to make sure that a professional or another representative for the patient or family talk to them before and after the deliberation in the group, so that their views and experiences are described as well as possible, and that they get feedback. A third possibility is having a representative from a patient or family organisation as a permanent member of the group.

There is sparse research on patient and family involvement in clinical ethics support. In one study on Norwegian ethics committees, the relatives were generally very positive to being included in discussions [65]. However, this was a qualitative study from somatic health care, and deliberations in clinical ethics committees are not the same as ethics reflections groups.

Another study evaluates patient- and client participation in two different series of moral case deliberation (MCD) [63]. In one of the groups, patient participation was required by adding one member of the client council to an already existing MCD group of healthcare professionals. In the other group, they started from the beginning with an equal mix of members from the client council, the family council and the team of healthcare professionals. The second group evaluated client participation more positively. However, the researchers conclude that client participation ‘requires continuous reflection and alertness on relational dynamics and the quality of and conditions for dialogue. Patient and family participation puts the essentials of MCD (i.e. dialogue) to the test’ [ibid., p. 207,16]. Therefore, work on how to systematically integrate patients and family in ethics reflection, in both dealing with ethical challenges and in the way coercion is being used, is an important task for future practice and research.

## Conclusion

In order to provide good treatment and care in the context of coercion, it is important that healthcare professionals have continuous attention to what good treatment and care is, and what it means to be a good professional and a good organisation. In conclusion, health care professionals in this project are satisfied with systematic ethics reflection related to the use of coercion. According to the participants in the present study, ethics reflection groups not only had positive effects on the dilemmas on coercion dealt with in the groups, but also on other aspects of their work, like teamwork and multidisciplinary cooperation, awareness of informal coercion, the coercive culture, attitudes towards the patient, and on patient and family involvement. Systematic ethics reflection made a difference for many participants in this project by helping them to develop a new language, which described more accurately the ethical challenges they were facing. Furthermore, the employees helped each other develop new perspectives and horisons related to the use of coercion, good treatment and care, and good cooperation.

This study confirms the potential “of creating and facilitating a moral space within the institution that encourages critical, reflective and collaborative moral thinking” (14), at the same time realising that “keeping moral space open”, as Walker [66] puts it, is an ongoing process requiring a consistent and a long-term perspective. The theoretical foundation of systematic ethics reflection – discourse ethics and hermeneutics – contributes to keeping the moral space open, and being sensitive to both the often implicit or hidden moral dimension of everyday work, and how presuppositions of what is taken for granted or seen as necessary or morally good can be deconstructed and challenged, in order to stimulate free and critical thinking.

The present study has shown that systematic ethics reflection in the health services is a young discipline with great potential. In the future, it will be important to develop the work of systematic ethics reflection so that exploration of healthcare challenges includes all affected parties, patients, relatives as well as employees.

## Abbreviations

CME: Centre for Medical Ethics (University of Oslo)
PET-project: the English name for ‘PET’ is ‘Mental health care, Ethics and Coercion’
